# Mental health: A cause or consequence of injury? A population-based matched cohort study

**DOI:** 10.1186/1471-2458-6-114

**Published:** 2006-05-02

**Authors:** Cate M Cameron, David M Purdie, Erich V Kliewer, Rod J McClure

**Affiliations:** 1 School of Medicine, Griffith University, Meadowbrook, Australia; 2 Queensland Institute of Medical Research, Brisbane, Australia; 3 Department of Epidemiology and Cancer Registry, CancerCare Manitoba, Winnipeg, Canada; 4 Department of Community Health Sciences, University of Manitoba, Winnipeg, Canada; 5 School of Public Health, University of Sydney, Sydney, Australia

## Abstract

**Background:**

While a number of studies report high prevalence of mental health problems among injured people, the temporal relationship between injury and mental health service use has not been established. This study aimed to quantify this relationship using 10 years of follow-up on a population-based cohort of hospitalised injured adults.

**Methods:**

The Manitoba Injury Outcome Study is a retrospective population-based matched cohort study that utilised linked administrative data from Manitoba, Canada, to identify an inception cohort (1988–1991) of hospitalised injured cases (ICD-9-CM 800–995) aged 18–64 years (n = 21,032), which was matched to a non-injured population-based comparison group (n = 21,032). Pre-injury comorbidity and post-injury mental health data were obtained from hospital and physician claims records. Negative Binomial regression was used to estimate adjusted rate ratios (RRs) to measure associations between injury and mental health service use.

**Results:**

Statistically significant differences in the rates of mental health service use were observed between the injured and non-injured, for the pre-injury year and every year of the follow-up period. The injured cohort had 6.56 times the rate of post-injury mental health hospitalisations (95% CI 5.87, 7.34) and 2.65 times the rate of post-injury mental health physician claims (95% CI 2.53, 2.77). Adjusting for comorbidities and pre-existing mental health service use reduced the hospitalisations RR to 3.24 (95% CI 2.92, 3.60) and the physician claims RR to 1.53 (95% CI 1.47, 1.59).

**Conclusion:**

These findings indicate the presence of pre-existing mental health conditions is a potential confounder when investigating injury as a risk factor for subsequent mental health problems. Collaboration with mental health professionals is important for injury prevention and care, with ongoing mental health support being a clearly indicated service need by injured people and their families. Public health policy relating to injury prevention and control needs to consider mental health strategies at the primary, secondary and tertiary level.

## Background

Previous research suggests that psychosocial and mental health sequelae are some of the most disabling consequences of injury [1-4]. However, few studies have considered the presence of pre-existing mental health conditions as a potential confounder when investigating injury as a risk factor for subsequent mental health problems. While a number of studies report high prevalence of psychiatric disorders among injured people [5-7], few comprehensively examine the temporal relationship between injury and mental health conditions [4,8]. Most outcome studies focus on post-injury variables with limited attention given to the effect of pre-injury factors, particularly with regards to pre-injury health status and pre-existing mental health conditions [9].

Conclusions from previous studies that have described the prevalence of post-injury mental health have been limited by the study methods used. These include small sample sizes [1,8], non population-based sampling [1,10] and the lack of population comparison groups [10,11]. Most are injury type specific [6,12] or injury severity specific [6,9], with the length of follow-up rarely beyond 5 years [6,7].

The aim of this paper was to quantify the relationship between injury and mental health service use for 10 years post-injury event, controlling for demographic factors and pre-existing comorbidities, including pre-existing psychiatric conditions. Clarification of mental health conditions as a risk factor, potential confounder and outcome of injury is essential for improving injury prevention strategies and post-injury care.

## Methods

### Study design and setting

The Manitoba Injury Outcome Study is a population-based retrospective matched cohort study using linked administrative health data from the province of Manitoba, Canada [13,14]. The study was approved by the University of Manitoba Research Ethics Board, Manitoba Health's Health Information Privacy Committee and the University of Queensland Ethics Committee, Australia.

### Study participants

The injured cohort included all persons aged 18–64 years, resident in the province of Manitoba, who were hospitalised with an injury between 1 January 1988 and 31 December 1991 (n = 21,032). Injury related hospitalisations were identified using the *International Classification of Diseases, Ninth edition, Clinical Modification *(ICD-9-CM) codes 800–995 (excluding late effects from injury 905–909 and allergies from within 995), in the first or second diagnostic fields of the hospital record. The first injury-related hospital record was designated as the index injury.

A non-injured comparison cohort was randomly selected from the total remaining province population, identified using the Manitoba population registry, matched on Aboriginal status, age, gender and geographic location of residence of the injured case. The health claims databases and population registry can be linked using unique identification numbers. Residents of personal care homes (PCH), patients in extended hospital care, and persons not resident in the province for 12 months prior to the admission date of the index record, were excluded.

### Injury classification

The injured cohort was analysed by the nature of injury codes (ICD-9-CM 800–995), and subgroup comparisons were made with the matched comparison group. Seven subgroups were created across ICD subchapter headings (brain injury, spinal injury, burns, long bone fractures, poisonings, internal injuries and other). Injury Severity Scores (ISS) were generated by ICDMAP-90© software from Johns Hopkins University. An ISS of 16 or greater was considered to be a major injury, an ISS of 9–15 a moderate injury, and a mild injury was defined as an ISS of 1–8 [15,16]. Not all injured cases were severity scored as ICDMAP-90© maps only a proportion of the total Injury and Poisonings ICD-9-CM codes.

### Pre-injury health service use and comorbidity measures

For both the injured and non-injured cohorts, frequency and types of pre-existing conditions were identified from their health service use records during the 12-months prior to the index injury [14]. In addition, two levels of comorbidity severity were defined for three disease categories (musculoskeletal conditions, mental health conditions and previous injuries/poisonings). A 'mild condition' was one which involved one to three ambulatory physician claims and no hospital separations; a 'moderate-severe condition' was defined as four or more ambulatory physician claims or at least one hospitalisation for that condition. Individuals were coded as not having a condition in circumstances where they had no health services contact.

Pre-existing comorbidity for the two cohorts was also quantified using the Dartmouth-Manitoba version of the Charlson Comorbidity Index (CCI) [17]. The CCI was developed in 1987 as a method of classifying comorbid conditions. Further modifications were developed by Dartmouth-Manitoba collaboration that included some diagnoses that were not in the original CCI [18,19]. The total comorbidity score is the cumulative increased likelihood of one-year mortality [19]. The greater the CCI score, the more severe the burden of comorbidity [17].

### Outcome measures

Two outcome variables were used to explore long-term post-injury mental health service use – counts of post-injury mental health related hospital separations and ambulatory physician claims (excluding emergency department presentations). All hospital records and physician claims with a primary diagnosis of ICD-9-CM 'Mental Disorders' codes (290–319) were identified for the 10 year period following the date of the index injury. To identify patterns of post-injury mental health service use, the hospital and physician claims data were further separated into the ICD-9-CM subchapter codes for mental disorders.

### Calculation of person-years (PYs) at risk

Using the population registry information, the total time a person was alive, living in Manitoba and eligible for health coverage, was calculated for the 10 years following the index injury date.

### Analysis

Statistical significance of differences between injured and non-injured cohorts for pre-injury rates of health service use was assessed by chi-squared test for categorical data and with the Mann-Whitney U test for continuous data because of non-normal distributions. All tests were two sided with a 5% level of significance.

Negative Binomial regression was used to estimate crude and adjusted rate ratios (RRs) between exposure (injury) and outcome (mental health service use) [20,21]. Those factors shown to be associated with both the exposure and the outcome in univariate analysis were included in the model as potential confounders. Matching variables were included in the model, as it has been shown where matched-cohort members have different lengths of follow-up, confounding by matching variables may occur over time [22]. The final model included age, gender, place of residence; CCI, pre-injury cumulative hospital length of stay (LOS), pre-injury physician claims; generated scores for pre-existing mental health conditions, musculoskeletal conditions and previous injuries.

To identify patterns in post-injury mental health service use, the hospital and physician claims data were categorised into the ICD-9-CM subchapter codes for mental disorders.

Analysis was completed using SAS version 8.2.

## Results

### Characteristics of the injured cohort

In total, 21,032 injured cases were identified. Most injuries occurred in males (64%) and in the younger age categories 18–34 years (54%). Approximately 40% of cases lived in urban regions, 40% in rural areas and the remaining 20% resided in remote northern areas of the province. 'Other' accidents 28.5%, accidental falls 22.9%, transport related 18.0%, and homicide or injury inflicted by others 10.9% accounted for the majority of the external causes. Further details of these characteristics have been published previously [14].

### Pre-injury health service use

The injured cohort had significantly more all-cause health service use in the 12 months *prior *to the injury event than their matched non-injured counterparts. Injured people had higher CCI scores, more hospitalisations, longer average length of stay in hospital, a greater number of physician claims and were more likely to have been admitted to hospital or seen a physician multiple times for a mental health condition, musculoskeletal condition or for a previous injury. Further details of these differences have been published previously [14].

Mental health conditions were the most frequent cause of hospitalisations for the injured cohort in the pre-injury period. The injured cohort had 9.31 times the rate of separations from hospital for a mental health condition in the 12 months prior to the injury than the non-injured cohort (95% CI 7.35, 11.77) (Table 1). Almost half of all these mental health related hospital separations were for alcoholic psychoses, affective psychoses and schizophrenic disorders. During this time period, injured people also had 3.50 times the rate of mental health physician claims than the non-injured (95% CI 3.42, 3.78) (Table 2). Over 80% of all pre-injury mental health physician claims were for 'non-psychotic or personality disorders', more specifically for panic, anxiety or depression.

### Loss to follow-up

Loss to follow-up in this study was minimal, with 10.9% in the injured cohort and 14% in the non-injured lost over the total 10 years. Of the injured cohort, 8% died during the 10 years, compared with 3.6% who died among the non-injured.

### Post-injury mental health service use

The injured cohort had higher rates of mental health service use every year of the study period, compared to the non-injured (Table 1 and Table 2). However, the greatest differences in rates of post-injury mental health service use between the two cohorts occurred during the first 12 months following the injury (mental health hospitalisations unadjusted RR = 8.86, mental health physician claims unadjusted RR = 3.86).

Rates of post-injury mental health hospitalisations (Table 3) and mental health physician claims (Table 4) increased in both cohorts as the presence and severity of pre-existing mental health conditions increased. However, there was no difference in the adjusted RRs between the three levels of pre-existing mental health severity. The injured cohort had approximately three times the rate of post-injury mental health hospitalisations and almost 1.5 times the rate of post-injury mental health physician claims when compared with the non-injured cohort, regardless of the level of pre-existing mental health severity.

Adjusting for demographic factors, prior comorbidities, including pre-existing mental health conditions, decreased the overall 10 year RR for post-injury mental health hospitalisations from 6.56 to 3.24 (95% CI 2.92, 3.60) (Table 1) and reduced the RR for post-injury mental health physicians claims from 2.65 to 1.53 (95% CI 1.47, 1.59) (Table 2). Despite this reduction, the adjusted post-injury RRs remained significantly elevated for all years of follow-up for both hospitalisations and physician claims.

Excluding cases of self-harm from the injured cohort had limited effect on the differences in mental health service use between the injured and comparison cohort. All RRs remained statistically significant for post-injury mental health hospitalisations (adjusted RR = 2.88, 95% CI 2.56, 3.23) and post-injury mental health physician claims (adjusted RR = 1.44, 95% CI 1.38, 1.50).

### Post-injury mental health service use by injury type and severity

Rates of post-injury mental health hospitalisations and physicians claims varied across different injury types (Table 5 and Table 6). After adjusting for confounders, the burns group were 6.70 times more likely to be hospitalised post-injury for a mental health condition than their non-injured counterparts (95% CI 2.52, 17.76), followed by poisonings (RR = 5.74, 95% CI 4.48, 7.35). Poisoning cases had the highest rate of physicians claims compared to the non-injured (RR = 2.43, 95% CI 2.18, 2.72). The greatest change from crude to adjusted RRs occurred in the poisonings group, due to the high prevalence of self-harm injuries and pre-existing mental health conditions. Over 80% of all self-harm injuries in the total cohort occurred in the poisonings group.

Adjusted RRs for post-injury mental health hospitalisations during follow-up showed some increase as the severity of the injury increased (Table 5). The minor cases were 2.60 times more likely to be hospitalised for a mental health condition during the follow-up compared to the comparison cohort. The adjusted RR increased to 3.14 for the moderately injured to 3.86 for the most severely injured. Across all levels of severity the injured cohort was more likely to have seen a physician for a mental health condition than the non-injured, with the greatest difference occurring in the most severely injured cases (Table 6).

### Types of post-injury mental health service use (ICD-9-CM subchapters)

The injured cohort had 20.3 times the number of post-injury hospitalisations and 4.2 times the number of physician claims for personality disorders than the comparison cohort. Almost eight times more post-injury hospitalisations in the injured cohort were related to alcohol or drugs, compared with the non-injured. Relative differences for each of the other mental health subchapter types were of a fourfold magnitude.

While the injured cohort had greater numbers of mental health hospitalisations and physician claims, the proportion of service use for particular mental health conditions was similar in both cohorts. Almost half of all post-injury mental health hospitalisations were for non-psychotic or personality disorders (injured 49.3% and non-injured 46.8%), a third of all post-injury mental health hospitalisations had a primary diagnosis of affective and delusional psychoses (injured 31.5% and non-injured 32.8%) and the remaining 20% were for organic psychoses. The majority of mental health physician claims were for non-psychotic or personality disorders (injured 79.2% and non-injured 86.2%), followed by claims for affective and delusional psychoses (injured 18.0% and non-injured 12.1%). Of all the injury types, poisonings cases were most likely to have subsequent personality disorder diagnoses.

## Discussion

The current study aimed to describe and quantify the relationship between injury and subsequent mental health service use, as well as the contribution of pre-existing mental health conditions on post-injury mental health service use. We found an increased risk of mental health service use for at least 10 years following the injury event. While the greatest difference in mental health service use between the injured and non-injured occurred during the year prior to the injury event, statistically significant differences remained for the 10 years following injury, even after adjusting for demographic characteristics and pre-injury health status, including pre-existing psychiatric conditions. While there was a marked increase in health service use for alcohol and drug related conditions and personality disorders in the injured cohort, the proportional distributions of all mental health conditions were similar in both cohorts.

The study that was most similar in methods to the current study was the one undertaken by Dryden et al in 2004 [23], who examined cases of spinal cord injury (SCI) and a matched non-injured group using similar linked Canadian provincial health data. Their study found that cases with SCI were more than twice as likely to be treated for depression during the six years of follow-up than were the non-injured (RR = 2.54, 95% CI 1.95, 3.31). Cases were also more likely to be treated by a psychiatrist and more likely to be to be admitted to hospital with a mental health condition than the comparison group. However, Dryden et al [23] did not examine the temporal relationship between the injury and psychiatric disorders, nor control for pre-existing mental health conditions in the analysis that compared the injured and non-injured subjects.

While the present study did not specifically aim to identify risk factors for injury, the observed pre-injury rates of mental health service use suggest that the presence of mental health conditions may be associated with an increased risk of injury. This finding is consistent with previous studies that have attempted to measure the prevalence of pre-injury mental health conditions in samples of injured people and compare it to population norms. Patterson et al [9], found the rates of pre-existing mental health symptoms in a sample of adult burn patients were significantly higher than that of a national normative sample on Rand Mental Health Inventory scores. Principal mechanisms underlying the etiologic link between mental health conditions and injury relate to risk taking behaviours, alcohol misuse, cognitive impairments, medication use and self-harming ideation [24-26].

In the current study, over and above pre-existing conditions and those of self-harm, injury appeared to be associated with a threefold increase in mental health hospitalisations and 1.4 times the number of mental health physician claims in the decade following the injury. These data support the results of other studies which found pre-existing mental health conditions as both a risk factor for injury and a confounder of the relationship between injury and poor mental health outcomes following injury [8,27].

While there is a plausible causal pathway linking injury and post-injury mental health episodes of depression, anxiety or alcohol use, it is not obvious why injury would cause a personality disorder. The effect was likely to be due to the injury triggering a pre-existing latent personality disorder or by an increased rate of diagnosis through the increased contact with the health care system [28].

The use of a non-injured comparison group is a key element for attributing effects which have occurred a considerable time after the exposure [22]. While, to some extent, confounding by factors other than pre-existing morbidity was addressed by the matched study design, some unmeasured potential confounders remain. These include aspects of socio-economic status, education, risk taking and health behaviours associated with both the injury and outcome, over and above the matched variables, which were not included in the administrative datasets used in the analysis. Accordingly, the observed mental health service use that was attributed to the injury may have been overestimated.

When considering these data sources for epidemiological research, it is important to recognise the limitations and constraints of using administrative data that were primarily developed for accounting purposes. The Manitoba data have been used extensively in health services and epidemiological research by a number of different groups and there has been a strong emphasis on improving data quality [29-31]. While the quality of Manitoba health data is considered high with minimal errors, the potential for error must be acknowledged [18,31,32].

While administrative data lack details of individual risk factors, previous studies conducted in this population support the validity of deriving proxy measures of health status from health service use data, though the limitations of this practice have been acknowledged [31,33]. Some confidence in the pre-existing mental health estimates is warranted, given that the prevalence of mental health conditions in the non-injured comparison group was 9.24%, which is similar to the 10.4% estimated prevalence for any mental health condition or substance use disorder reported in the 2002 Canadian Community Health Survey [34].

## Conclusion

This study demonstrated that confounding factors, including pre-existing mental health conditions, accounted for almost half the mental health service use attributable to injury. However, after adjusting for confounding factors, the injured cases continued to have a significantly increased risk of post-injury mental health service use compared to the non-injured. Separating the consequences of injury for those with pre-existing mental health problems from those without pre-existing mental health problems is important because the effects of injury were two-fold: 1) injuries increased mental health service use for those with pre-existing mental health problems and 2) injuries lead to mental health service use among those without pre-injury mental health conditions. These findings indicate the presence of pre-existing mental health conditions is a potential confounder when investigating injury as a risk factor for subsequent mental health problems. Collaboration with mental health professionals is important for injury prevention and care, with ongoing mental health support being a clearly indicated service need by injured people and their families. Public health policy relating to injury prevention and control needs to consider mental health strategies at the primary, secondary and tertiary level.

## Abbreviations

CI, confidence interval; ICD-9-CM, *International Classification of Diseases*, Ninth edition, Clinical Modification; ISS, Injury Severity Score; LOS, length of stay; PCH, personal care home; PYs, person-years; RR, rate ratio; SCI, spinal cord injury.

## Competing interests

The author(s) declare that they have no competing interests.

## Authors' contributions

CMC conceived the study, participated in its design, conducted the data collection, analysis and manuscript preparation. DVP, EVK and RJM all directly participated in the research conception, design and execution; preparation of the manuscript; and approved the final version submitted.

## Financial support

This research was conducted, in part, at the School of Population Health, University of Queensland and was financially supported by the Centre of National Research on Disability and Rehabilitation Medicine, University of Queensland.

## Pre-publication history

The pre-publication history for this paper can be accessed here:



## Figures and Tables

**Table 1:** Rates of mental health hospitalisations for injured and non-injured cohorts, with rate ratios and 95% confidence intervals, by time periods

	**Number of Mental Health Hospitalisations**							
**Time Period**	**Injured** **(N = 21,032)**		**Non-Injured** **(N = 21,032)**		**Unadjusted Rate Ratio**	**95% Confidence Interval**	Adjusted Rate Ratio *	**95% Confidence Interval**
	**Hosp**	**Rate** **/10 PYs**	**Hosp**	**Rate** **/10 PYs**				
***Year prior***	*884*	*0.42*	*95*	*0.05*	*9.31*	*7.35–11.77*	*na*	*na*
**Year 1**	939	0.46	106	0.05	8.86	7.09–11.08	3.99	3.18–5.01
**Year 2**	674	0.34	106	0.05	6.27	4.95–7.96	2.88	2.27–3.66
**Year 3**	641	0.33	88	0.05	7.23	5.62–9.29	3.63	2.82–4.68
**Year 4**	590	0.31	96	0.05	6.21	4.84–7.96	3.21	2.49–4.12
**Year 5**	588	0.31	86	0.05	6.94	5.34–9.01	3.77	2.90–4.90
**Year 6**	546	0.30	120	0.07	4.53	3.57–5.75	2.56	2.02–3.25
**Year 7**	515	0.29	90	0.05	5.82	4.49–7.55	2.98	2.29–3.87
**Year 8**	550	0.31	97	0.05	5.68	4.37–7.39	2.66	2.05–3.46
**Year 9**	496	0.29	92	0.05	5.47	4.18–7.15	2.91	2.22–3.81
**Year 10**	495	0.31	76	0.05	6.57	4.92–8.77	3.45	2.58–4.62
**Total**	**6,034**	**0.33**	**957**	**0.05**	**6.56**	**5.87–7.34**	**3.24**	**2.92–3.60**

* Adjusted for age, gender, place of residence, and comorbidities in 12 months prior to injury date (including CCI, cumulative LOS, number of physician claims, pre-existing psychiatric condition, pre-existing musculoskeletal condition and previous injuries)

**Table 2:** Rates of mental health physician claims for injured and non-injured cohorts, with rate ratios and 95% confidence intervals, by time periods

	**Number of Mental Health Physician Claims**							
**Time Period**	**Injured** **(N = 21,032)**		**Non-Injured** **(N = 21,032)**		**Unadjusted Rate Ratio**	**95% Confidence Interval**	Adjusted Rate Ratio *	**95% Confidence Interval**
	**Claims**	**Rate** **/10 PYs**	**Claims**	**Rate** **/10 PYs**				
***Year prior***	*20,642*	*9.81*	*5,899*	*2.80*	*3.50*	*3.42–3.78*	*na*	*na*
**Year 1**	23,185	11.25	6,044	2.96	3.86	3.59–4.16	2.08	1.95–2.21
**Year 2**	18,913	9.46	6,274	3.19	2.97	2.75–3.21	1.62	1.51–1.73
**Year 3**	17,143	8.81	6,217	3.23	2.74	2.53–2.96	1.55	1.45–1.66
**Year 4**	16,343	8.58	6,598	3.49	2.47	2.29–2.67	1.46	1.36–1.57
**Year 5**	14,903	7.97	6,267	3.36	2.38	2.20–2.57	1.48	1.38–1.59
**Year 6**	14,481	7.88	6,480	3.53	2.24	2.07–2.42	1.47	1.37–1.58
**Year 7**	14,613	8.10	6,552	3.62	2.24	2.07–2.42	1.49	1.39–1.61
**Year 8**	15,074	8.51	7,173	4.02	2.12	1.96–2.29	1.36	1.27–1.47
**Year 9**	14,961	8.60	7,167	4.07	2.12	1.96–2.28	1.34	1.24–1.44
**Year 10**	13,849	8.68	6,887	4.25	2.02	1.87–2.19	1.29	1.19–1.39
**Total**	**163,465**	**8.82**	**65,659**	**3.55**	**2.65**	**2.53–2.77**	**1.53**	**1.47–1.59**

* Adjusted for age, gender, place of residence, and comorbidities in 12 months prior to injury date (including CCI, cumulative LOS, number of physician claims, pre-existing psychiatric condition, pre-existing musculoskeletal condition and previous injuries)

**Table 3:** Rates of post-injury mental health hospitalisations, for injured and non-injured cohorts, with rate ratios and 95% confidence intervals, by severity of pre-existing mental health condition

**Pre-existing Mental Health Condition**	**Injured**			**Non-Injured**			**Unadjusted Rate Ratio**	**95% Confidence Interval**	Adjusted Rate Ratio *	**95% Confidence Interval**
	**N**	**Hosp**	**Rate** **/10 PYs**	**N**	**Hosp**	**Rate** **/10 PYs**				
**No condition **^a^	16,984	1,778	0.12	19,089	551	0.03	3.67	3.19–4.23	3.25	2.81–3.76
**Mild condition **^b^	2,550	1,018	0.46	1,520	175	0.13	3.59	2.80–4.61	3.19	2.46–4.12
**Moderate-severe**^c^	1,498	3,238	2.61	423	231	0.62	4.11	3.20–5.28	3.12	2.42–4.01
**Total**	**21,032**	**6,034**	**0.33**	**21,032**	**957**	**0.05**	**6.56**	**5.87–7.34**	**3.24**	**2.92–3.60**

* Adjusted for age, gender, place of residence, and comorbidities in 12 months prior to injury date (including CCI, cumulative LOS, number of physician claims, pre-existing musculoskeletal condition and previous injuries)

^a ^No admissions or physician claims for that condition in pre-injury period

^b ^1–3 physician claims and no admissions for that condition in pre-injury period

^c ^1 or more admissions and/or 4 or more physician claims for that condition in pre-injury period

**Table 4:** Rates of post-injury mental health physician claims, for injured and non-injured cohorts, with rate ratios and 95% confidence intervals, by severity of pre-existing mental health condition

**Pre-existing Mental Health Condition**	**Injured**			**Non-Injured**			**Unadjusted Rate Ratio**	**95% Confidence Interval**	Adjusted Rate Ratio *	**95% Confidence Interval**
	**N**	**Claims**	**Rate** **/10 PYs**	**N**	**Claims**	**Rate** **/10 PYs**				
**No condition **^a^	16,984	54,383	3.60	19,089	36,936	2.20	1.69	1.61–1.77	1.56	1.49–1.64
**Mild condition **^b^	2,550	30,385	13.79	1,520	12,932	9.59	1.46	1.33–1.61	1.36	1.23–1.50
**Moderate-severe**^c^	1,498	78,697	63.41	423	15,791	42.50	1.56	1.37–1.78	1.49	1.30–1.71
**Total**	**21,032**	**163,465**	**8.82**	**21,032**	**65,659**	**3.55**	**2.65**	**2.53–2.77**	**1.53**	**1.47–1.59**

* Adjusted for age, gender, place of residence, and comorbidities in 12 months prior to injury date (including CCI, cumulative LOS, number of physician claims, pre-existing musculoskeletal condition and previous injuries)

^a ^No admissions or physician claims for that condition in pre-injury period

^b ^1–3 physician claims and no admissions for that condition in pre-injury period

^c ^1 or more admissions and/or 4 or more physician claims for that condition in pre-injury period

**Table 5:** Rates of mental health hospitalisations for injured and non-injured cohorts, with rate ratios and 95% confidence intervals, by injury type and injury severity

			**Number of Mental Health Hospitalisations**									
**Injury Types and Severity Level**			**Injured** **(N = 21,032)**			**Non-Injured** **(N = 21,032)**			**Unadjusted Rate Ratio**	**95% Confidence Interval**	Adjusted Rate Ratio *	**95% Confidence Interval**
	**N**	**%**	**Hosp**	**%**	**/100 PYs**	**Hosp**	**%**	**/100 PYs**				
**Injury Subgroup**												
**Brain injury**	1,290	6.1	260	4.3	2.44	89	9.3	0.79	3.16	2.03–4.92	2.82	1.84–4.33
**Spinal injury**	95	0.5	2	0.0	0.24	1	0.1	0.12	3.45	0.07–155.69	–	–
**Burns**	524	2.5	150	2.5	3.25	14	1.5	0.31	9.93	4.12–23.94	6.70	2.52–17.76
**Fracture of long bones**	2,515	12	362	6.0	1.63	111	11.6	0.50	3.41	2.36–4.91	2.62	1.82–3.76
**Poisonings**	2,169	10.3	2,358	39.1	12.68	140	14.6	0.72	18.72	14.75–23.76	5.74	4.48–7.35
**Internal injuries**	593	2.8	98	1.6	1.91	30	3.1	0.59	2.90	1.43–5.85	3.09	1.57–6.10
**All other injuries**	13,846	65.8	2,804	46.4	2.27	572	59.8	0.47	5.03	4.34–5.82	2.91	2.54–3.35
**Injury Severity Score (ISS)**												
**Minor (ISS 1–8)**	14,599	69.4	2,716	45.0	2.08	620	64.8	0.48	4.34	3.75–5.02	2.60	2.27–2.99
**Moderate (ISS 9–15)**	1,746	8.3	270	4.5	1.79	63	6.6	0.41	4.72	3.03–7.37	3.14	2.05–4.80
**Severe (ISS = 16)**	657	3.1	120	2.0	2.43	49	5.1	0.84	3.17	1.63–6.17	3.86	1.98–7.54
**No ISS Computed †**	4,030	19.2	2,928	48.5	8.45	225	23.5	0.63	14.43	11.86–17.57	5.07	4.17–6.15

* Adjusted for age, gender, place of residence, and comorbidities in 12 months prior to injury date (including CCI, cumulative LOS, number of physician claims, pre-existing psychiatric condition, pre-existing musculoskeletal condition and previous injuries)

^† ^ISS scores not computed for ICD-9-CM codes 930–939 foreign bodies; 958 complications; 960–979 poisonings; 980–989 toxic substances; 990–995 other.

**Table 6:** Rates of mental health physician claims for injured and non-injured cohorts, with rate ratios and 95% confidence intervals, by injury type and injury severity

			**Number of Mental Health Physician Claims**									
**Injury Types and Severity Level**			**Injured** **(N = 21,032)**			**Non-Injured** **(N = 21,032)**			**Unadjusted Rate Ratio**	**95% Confidence Interval**	Adjusted Rate Ratio *	**95% Confidence Interval**
	**N**	**%**	**Claims**	**%**	**/10 PYs**	**Claims**	**%**	**/10 PYs**				
**Injury Subgroup**												
**Brain injury**	1,290	6.1	7,509	4.6	7.05	3,760	5.7	3.32	2.18	1.80–2.60	1.92	1.63–2.27
**Spinal injury**	95	0.5	114	0.1	1.38	211	0.3	2.47	0.68	0.39–1.17	0.82	0.51–1.33
**Burns**	524	2.5	3,837	2.3	8.33	1,235	1.9	2.77	3.12	2.30–4.23	2.02	1.53–2.67
**Fracture of long bones**	2,515	12	13,409	8.2	6.04	7,530	11.5	3.40	1.82	1.60–2.07	1.27	1.12–1.43
**Poisonings**	2,169	10.3	56,689	34.7	30.48	9,273	14.1	4.79	7.04	6.28–7.89	2.43	2.18–2.72
**Internal injuries**	593	2.8	4,257	2.6	8.29	1,841	2.8	3.60	2.33	1.79–3.03	1.57	1.25–1.98
**All other injuries**	13,846	65.8	77,650	47.5	6.30	41,809	63.7	3.43	1.93	1.83–2.04	1.41	1.34–1.48
**Injury Severity Score (ISS)**												
**Minor (ISS 1–8)**	14,599	69.4	80,629	49.3	6.17	42,499	64.7	3.31	1.94	1.84–2.05	1.38	1.31–1.44
**Moderate (ISS 9–15)**	1,746	8.3	8,009	4.9	5.32	5,515	8.4	3.60	1.49	1.28–1.73	1.24	1.09–1.43
**Severe (ISS = 16)**	657	3.1	4,620	2.8	9.35	1,861	2.8	3.20	3.28	2.55–4.22	2.58	2.04–3.26
**No ISS Computed **^†^	4,030	19.2	70,207	42.9	20.27	15,784	24.0	4.42	5.05	4.60–5.54	2.03	1.87–2.21

* Adjusted for age, gender, place of residence, and comorbidities in 12 months prior to injury date (including CCI, cumulative LOS, number of physician claims, pre-existing psychiatric condition, pre-existing musculoskeletal condition and previous injuries)

^† ^ISS scores not computed for ICD-9-CM codes 930–939 foreign bodies; 958 complications; 960–979 poisonings; 980–989 toxic substances; 990–995 other.
